# Altered Resting-State Functional Networks in Nondialysis Patients with Stage 5 Chronic Kidney Disease: A Graph–Theoretical Analysis

**DOI:** 10.3390/brainsci13040628

**Published:** 2023-04-06

**Authors:** Lijun Song, Xu Liu, Wenbo Yang, Qian Chen, Han Lv, Zhenghan Yang, Wenhu Liu, Hao Wang, Zhenchang Wang

**Affiliations:** 1 Department of Radiology, Beijing Friendship Hospital, Capital Medical University, No. 95 Yong An Road, Beijing 100050, China; 2 Department of Nephrology, Beijing Friendship Hospital, Capital Medical University, No. 95 Yong An Road, Beijing 100050, China

**Keywords:** CKD5 ND, brain network, graph theory, cognitive disorder, hemodialysis

## Abstract

This study aimed to investigate the topological characteristics of the resting-state functional network and the underlying pathological mechanism in nondialysis patients with stage 5 chronic kidney disease (CKD5 ND). Eighty-five subjects (21 patients with CKD5 ND, 32 patients with CKD on maintenance hemodialysis (HD), and 32 healthy controls (HCs)) underwent laboratory examinations, neuropsychological tests, and brain magnetic resonance imaging. The topological characteristics of networks were compared with a graph–theoretical approach, and correlations between neuropsychological scores and network properties were analyzed. All participants exhibited networks with small-world attributes, and global topological attributes were impaired in both groups of patients with CKD 5 (ND and HD) compared with HCs (*p* < 0.05); these impairments were more severe in the CKD5 ND group than in the HD group (*p* < 0.05). Compared with the HC group, the degree centrality of the CKD5 ND group decreased mainly in the basal ganglia and increased in the bilateral orbitofrontal gyrus, bilateral precuneus, and right cuneus. Correlation analysis showed that the degree of small-worldness, normalized clustering coefficients, and Montreal Cognitive Assessment (MoCA) scores were positively correlated and that characteristic path length was negatively correlated with these variables in patients with CKD5 ND. The nodal efficiency of the bilateral putamen (r = 0.53, *p* < 0.001 and r = 0.47, *p* < 0.001), left thalamus (r = 0.37, *p* < 0.001), and right caudate nucleus (r = 0.28, *p* = 0.01) was positively correlated with MoCA scores. In conclusion, all CKD5 ND patients exhibited changes in functional network topological properties and were closely associated with mild cognitive impairment. More interestingly, the topological property changes in CKD5 ND patients were dominated by basal ganglia areas, which may be more helpful to understand and possibly reveal the underlying pathological mechanisms of cognitive impairment in CKD5 ND.

## 1. Introduction

Chronic kidney disease (CKD) results in end-stage renal disease (ESRD), which leads to an impaired glomerular filtration rate (GFR < 15 mL/min/1.73 m^2^) [1,2]. CKD5 is defined as chronic renal failure resulting in less than 10% of renal function without receiving dialysis compared with that in the general population [3]. The prevalence of cognitive impairment (CI) is also high in nondialysis (ND) patients with stage 5 chronic kidney disease (CKD5 ND). Dialysis or renal transplant is the typical replacement therapy administered to patients with ESRD. Patients with CKD often have poor outcomes once hemodialysis (HD) is initiated, including high mortality within the first year and a wide range of clinical neurological and psychological complications (e.g., rapid cognitive and functional decline, stroke, and depression) that diminish their quality of life [4,5]. There has also been very limited success in improving CKD-related CI by targeting neuroinflammatory mechanisms and nutritional interventions [2]. Therefore, understanding the neuropathological mechanisms of CI in CKD5 ND patients and screening for potential biological markers associated with CKD5 ND may be important for finding new potential preventive and therapeutic tools.

Neuroimaging is useful for revealing the mechanisms underlying ESRD-related neurologic complications. Voxel-based morphometry [6] and diffusion tensor imaging [7] have been respectively used to investigate the brain volume and white matter integrity of ESRD patients. Arterial spin labeling (ASL) and quantitative susceptibility mapping (QSM) have also been used to investigate cerebral blood flow [8] and iron deposition [9] in the brains of ESRD patients. In addition, resting-state functional magnetic resonance imaging (rs-fMRI) has been widely used to explore the pathophysiological mechanism of cognitive dysfunction in ESRD patients. Recently, several rs-fMRI studies have indicated that ESRD patients undergoing maintenance HD exhibit decreased spontaneous brain activity in multiple brain regions, particularly in regions associated with the default mode network (DMN) [10,11,12,13]. Regional homogeneity (ReHo) values were decreased in multiple regions of the bilateral frontal, parietal, and temporal lobes in HD patients [13], accompanied by increased functional connectivity among these regions [14]. In addition, functional brain networks are disrupted in HD patients, and these alterations in brain topological features are associated with CI [15,16].

In recent years, increasing attention has been given to observations of resting-state brain activity [17]. Graph–theoretical analyses, which can identify changes in the topological properties of brain networks [18,19], have indicated disruptions in brain networks in patients with Alzheimer’s disease [20], schizophrenia [21], and diabetic nephropathy [22]. Although studies on brain injury in patients with ESRD are increasing, little is known about the alteration of the topological properties of brain networks in patients with CKD5 ND.

Therefore, in this study, we will initially explore the topological characteristics of functional brain networks in patients with CKD5 ND based on a graph–theoretical approach and make the following hypotheses: (1) CKD5 ND may exhibit disruption of global topological properties of the functional brain network; (2) node properties may exhibit abnormalities in patients with CKD5 ND; (3) altered topological characteristics of whole brain functional networks in patients with CKD5 ND correlate with MOCA scores.

## 2. Materials and Methods

### 2.1. Participants

**Figure 1:**
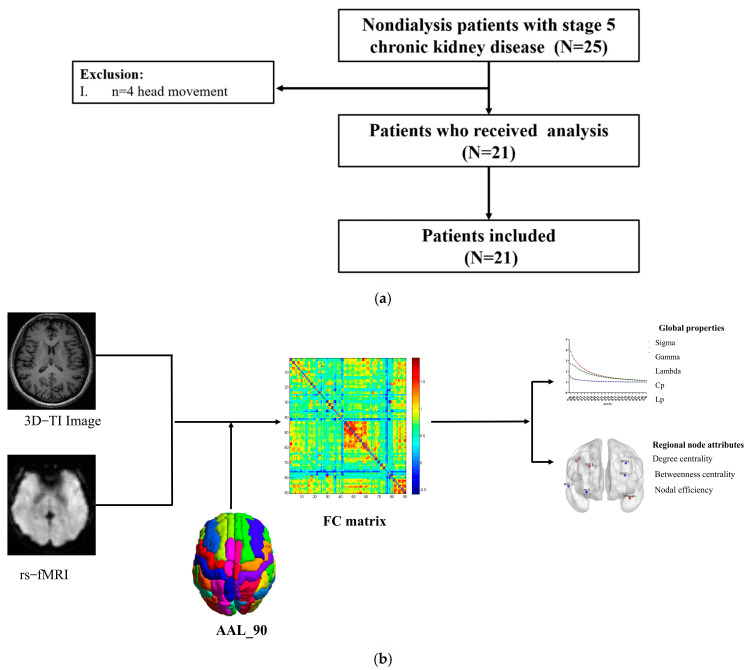
(**a**) Summary of nondialysis patients with stage 5 chronic kidney disease patient recruitment and exclusion. (**b**) Flowchart of the MRI signal analysis. AAL, anatomical automatic labeling.

In this study, patients in the Department of Nephrology of Beijing Friendship Hospital, Capital Medical University, and healthy controls (HCs) from the local community were recruited as subjects. In total, 21 patients with CKD5 ND (15 males and 6 females) and 32 patients with HD (19 males and 13 females) were recruited. Four patients with CKD5 ND were excluded because of head movement (Figure 1a). Thirty-two healthy subjects (12 males and 20 females) without any history of kidney disease served as HCs. All subjects were right-handed, and the HD patients had been on hemodialysis for at least 2 years before the study. The exclusion criteria were as follows: (1) other dialysis treatments; (2) a history of neuropathy; (3) neural diseases, including brain trauma, cerebrovascular disease, and brain tumor; (4) other systemic diseases; (5) any MRI contraindications; and (6) obvious head movement (≥3 mm/3°) during MRI. All subjects in this study signed informed consent forms.

### 2.2. Clinical Evaluations and Laboratory Examination

All patients with CKD5 ND underwent blood biochemistry and cognitive assessment before MRI scanning. Blood biochemical examinations included ferritin (ng/mL), urea (mmol/L), creatinine (µmol/L), albumin (g/L), calcium (mmol/L), phosphorus (mmol/L), hemoglobin (g/L), parathyroid hormone (pg/L), and serum C-reactive protein (mg/L). The Montreal Cognitive Assessment (MoCA) was used to evaluate the cognitive level of CKD5 ND and HD patients.

### 2.3. MRI Data Acquisition

All subjects underwent imaging using a 3.0-T magnetic resonance system (Discovery MR750w, General Electric, Milwaukee, WI, USA) and an eight-channel phased front coil. All subjects lay supine and were instructed to remain still. Sponge pads and earplugs were used to reduce head movement and scanner noise. The 3D T1-weighted structural images were obtained using a 3D-BRAVO sequence with the following parameters: slice thickness = 1 mm, no slice gap; 196 slices; repetition time (TR) = 8.492 ms; echo time (TE) = 3.276 ms; inversion time (TI) = 450 ms; field of view (FOV) = 24 × 24 cm^2^; flip angle (FA) = 15°; and matrix = 256 × 256. The rs-fMRI data were collected with the following parameters: slice thickness = 5 mm (1-mm slice gap); 28 slices; 200 time points; TR = 2000 ms; TE = 35 ms; FOV = 24 × 24 cm^2^; FA = 90°; matrix = 64 × 64; and acquisition time = 368 s.

### 2.4. Data Preprocessing

All functional images were preprocessed using the GRETNA software package (https://www.nitrc.org/projects/gretna/ accessed on 16 May 2022) [23]. The following steps were included in data preprocessing. (1) The first 5 volumes (out of a total of 200 volumes scanned for each subject) were removed. (2) Thus, a total of 195 volumes underwent slice-timing correction. (3) Realignment was performed to eliminate subjects with absolute head movement greater than 3 mm or 3°. (4) Spatial normalization to the standard Montreal Neurological Institute (MNI) template (resampled voxel size = 3 × 3 × 3 mm^3^) using DARTEL. [24] (5) The signal was detrended to remove linear and nonlinear drift or trends. (6) The nuisance signals (Friston-24 head motion parameters, white matter, and cerebrospinal fluid signals) were regressed by including them as covariables. (7) Bandpass filtering (0.01–0.08 Hz) was applied. (8) Scrubbing was used to deplete the volume of movement.

### 2.5. Functional Network Construction

The Gretna software package (https://www.nitrc.org/projects/gretna/ accessed on 16 May 2022) was used to construct a functional network for each subject. This network comprised nodes and edges, representing the functional connectivity between nodes. The nodes in the functional network were defined based on the automated anatomical labeling (AAL) template, which divides the whole brain (except the cerebellum) into 90 areas, including cortical and subcortical areas, each representing a node [25]. By extracting the average time series within each region of each subject and performing Pearson correlation analysis to calculate the correlation coefficient between each pair of nodes, a 90 × 90 correlation matrix was obtained for each subject. Subsequently, Fisher’s R-to-Z transformation was applied to the correlation matrix, and a series of sparse thresholds was used to convert the correlation matrix into a two-value network matrix [26].

### 2.6. Network Analysis

#### 2.6.1. Threshold Selection

We applied a series of sparsity ranges (0.05–0.5, step size: 0.01) for all correlation matrices. The minimum sparsity threshold ensured network connectivity, while the upper sparsity threshold ensured that the small-worldness of all functional networks was greater than 1. This sparsity range ensured that all the correlation matrices had a small-world nature and the same number of edges [27].

#### 2.6.2. Network Metrics

We calculated the global and node indicators under each sparsity threshold. Global indicators included the characteristic path length (Lp), clustering coefficients (Cp), normalized clustering coefficient (gamma), normalized characteristic path length (lambda), and small-worldness (sigma). The ratios of the Lp and Cp of the real network to the random network were defined as gamma and lambda, respectively (lambda = C/Cr, gamma = L/Lr). Small-worldness is defined as (C/Cr)/(L/Lr). With these definitions, a functional network is considered to exhibit “small-world” properties at gamma ≫ 1 and lambda ~1 or sigma > 1 [28]. Global efficiency (Eg) describes the ability of the network to process information in parallel, and local efficiency (Eloc) describes the fault tolerance of the network and, to a certain extent, reflects the ability of the network to handle random attacks. The node indicators included degree centrality, betweenness centrality, and node efficiency. Degree centrality describes the ability of the node to communicate information in the network. Node efficiency is used to measure the connectivity of the node with other nodes in the network and to examine the information transmission capacity of each node in the network, and betweenness centrality represents the influence of this node on information transfer between other nodes [29]. We calculated the area under the curve (AUC) of each indicator over the whole sparsity range.

### 2.7. Statistical Analysis

SPSS software was used for statistical analysis of demographic and clinical characteristics as well as the results of the neuropsychological tests. The chi-square test was used to analyze the group differences in categorical variables. An analysis of variance (ANOVA) or two-sample *t*-test was used to compare continuous variables. *p* < 0.05 was considered statistically significant.

Covariance analysis was used to determine group differences in the topological attributes of functional networks (small-worldness, global efficiency, and local efficiency). Node attributes were compared with independent-sample *t*-tests. 

## 3. Results

### 3.1. Demographic and Clinical Characteristics

Twenty-one patients with CKD5 ND, 32 HD patients, and 32 HCs were recruited for this study. The demographic and clinical features of the participants are presented in Table 1. There were no significant differences in age (*p* = 0.533), sex (*p* = 0.359), or education level (*p* = 0.283) among the three groups. Regarding the results of laboratory tests, there were significant differences in blood levels of urea, creatinine, hemoglobin, and Ca^2+^ between the CKD5 ND and HD groups (*p* < 0.05). Regarding the results of neuropsychological tests, the MoCA scores significantly differed between the CKD5 ND and HD groups (*p* < 0.05).

### 3.2. Alterations in Global Properties

**Figure 2:**
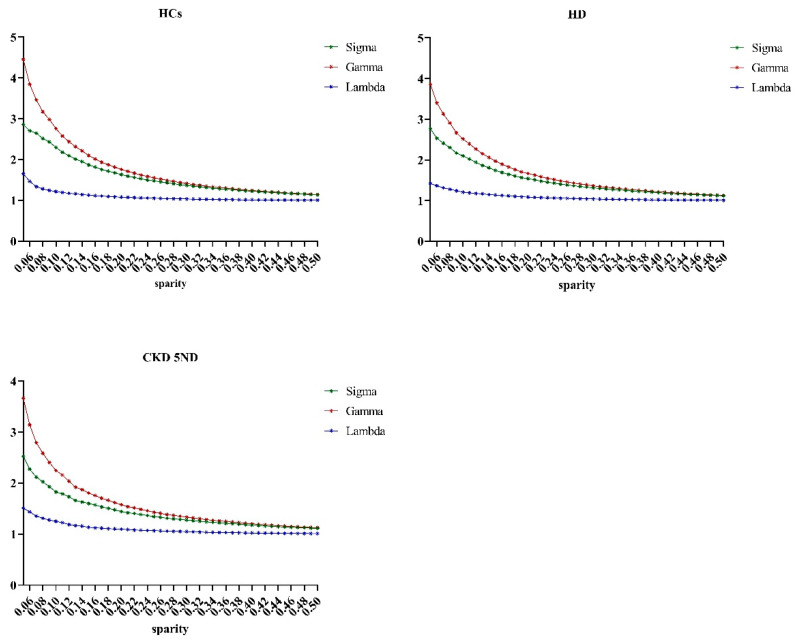
Within all defined sparsity thresholds (0.05–0.5), subjects in all three groups exhibited the small-world attributes, with the Gamma and Sigma values in all three groups greater than 1, and the Lambda value was approximately equal to 1, HCs, healthy controls; CKD5 ND, nondialysis patients with stage 5 chronic kidney disease; HD, hemodialysis patients. Sigma, small-worldness; Gamma, normalized clustering coefficient; Lambda, normalized clustering coefficient.

**Figure 3:**
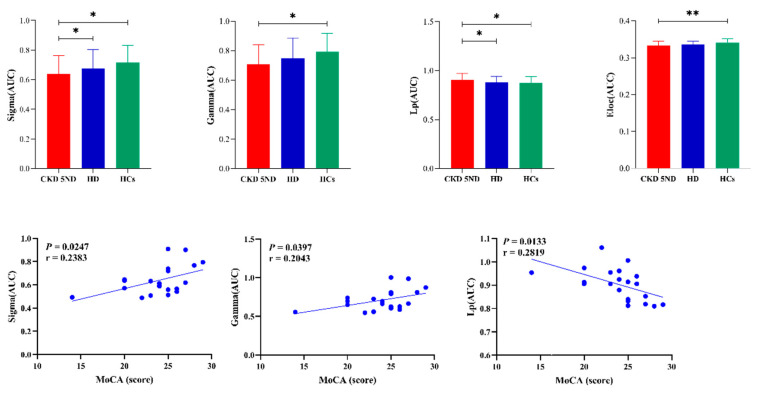
Comparisons of AUC values (thresholds ranged from 0.05 to 0.50) in small-world metrics (Sigma, Gamma, Lp) and Network Efficiency (Eloc) between the CKD5 ND, HD, and HC groups. (* *p* < 0.05; ** *p* < 0.01). AUC, the area under the curve; Sigma, small-worldness; Gamma, normalized clustering coefficient; Lp, characteristic path length; Eloc local efficiency.

Within the set sparsity range (0.05–0.5), all participants had networks that exhibited small-world attributes (sigma > 1, gamma > 1, lambda ≈ 1) (Figure 2). The sigma and gamma values decreased in the following order: the HC, HD, and CKD5 ND groups. The sigma values of the CKD5 ND group were significantly lower than those of the HC and HD groups (*p* < 0.0001). Patients with CKD5 ND also exhibited lower gamma values (*p* < 0.05) than HCs. The Lp values of the three groups increased in the following order: the HC, CKD5 ND, and HD groups. The Lp values were significantly higher in the patients with CKD5 ND than in the HC and HD groups (all *p* < 0.05). However, there were no significant differences in the Cp or lambda among the three groups (*p* > 0.05). In terms of network efficiency, the global and local efficiency were higher in the HC group than in the CKD5 ND group. The Eloc of patients with CKD5 ND was significantly lower than that of the HC group (*p* < 0.05), and the Eg was not significantly different among the three groups (Figure 3).

### 3.3. Alterations in Regional Node Attributes

**Figure 4:**
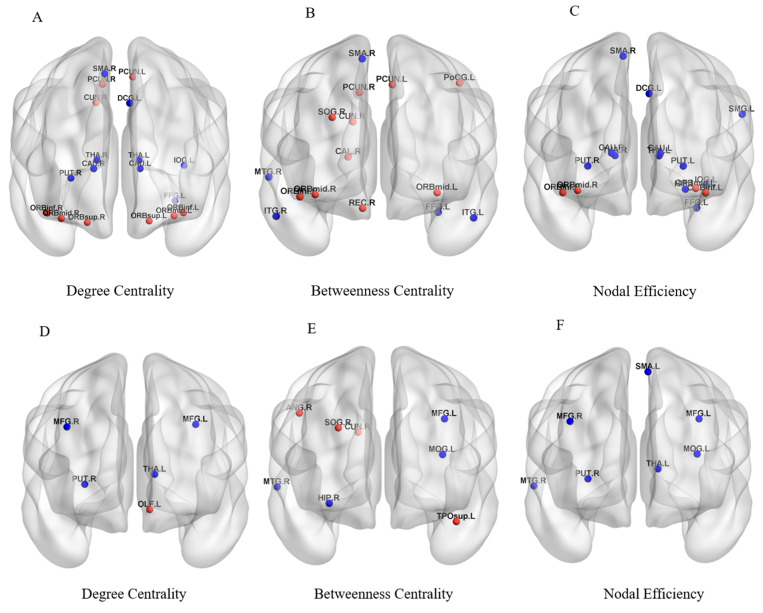
Brain regions with significant differences in the degree centrality (**A**), betweenness centrality (**B**), and nodal efficiency (**C**) among participants in the CKD5 ND and HC groups. Brain regions with significant differences in the degree centrality (**D**), betweenness centrality (**E**), and nodal efficiency (**F**) among participants in the CKD5 ND and HD groups. The red sphere demonstrated that nodal network properties in the CKD5 ND group were increased compared with the HC and HD groups. The blue sphere showed that the nodal network measures in the CKD5 ND group were decreased relative to the HC and HD groups.

Compared with the HC and HD groups, the degree centrality, betweenness centrality, and nodal efficiency were changed in some brain regions of patients with CKD5 ND (*p* < 0.05) (Figure 4 and Table 2). Compared with the HC group, patients with CKD5 ND displayed decreased degree centrality of the right supplementary motor area (SMA), left median cingulate and paracingulate gyri (DCG), left inferior occipital gyrus (IOG), left fusiform gyrus (FFG), bilateral caudate nucleus (CAU), right lenticular nucleus pars putamen (PUT), and bilateral thalamus (THA) and increased degree centrality of the bilateral orbital superior frontal gyrus (ORBsup), bilateral orbital middle frontal gyrus (ORBmid), bilateral orbital inferior frontal gyrus (ORBinf), bilateral precuneus (PCUN), and right cuneus (CUN) (Figure 4A, Table 2). Additionally, the CKD5 ND group showed decreased betweenness centrality of the right SMA, left FFG, right middle temporal gyrus (MTG), and bilateral inferior temporal gyrus (ITG) and increased betweenness centrality of the bilateral ORBmid, bilateral PCUN, right ORBinf, right gyrus rectus (REC), right calcarine fissure and surrounding cortex (CAL), right CUN, right superior occipital gyrus (SOG), and left postcentral gyrus (PoCG) (Figure 4B, Table 2). Moreover, the CKD5 ND group also exhibited decreased nodal efficiency of the right SMA, left DCG, left IOG, left FFG, bilateral CAU, bilateral PUT, bilateral THA, left hippocampus (HIP), and left supramarginal gyrus (SMG) and increased nodal efficiency of the bilateral ORBmid and bilateral ORBinf (Figure 4C, Table 2).

Compared with the HD group, patients with CKD5 ND displayed increased degree centrality of the left olfactory cortex (OLF) and decreased degree centrality of the bilateral MFG, right PUT, and left THA (Figure 4D, Table 2). Moreover, the CKD5 ND group also showed decreased betweenness centrality of the left MFG, right HIP, right MTG, and left middle occipital gyrus (MOG) and increased betweenness centrality of the right CUN, right SOG, left angular gyrus (ANG), and left superior temporal gyrus pars temporal pole (TPOsup) (Figure 4E, Table 2). In addition, the CKD5 ND group showed decreased nodal efficiency of the bilateral MFG, left SMA, left MOG, right PUT, left THA, and right MTG (Figure 4F, Table 2).

### 3.4. Relationships between Network Indicators and Clinical Variables

**Figure 5:**
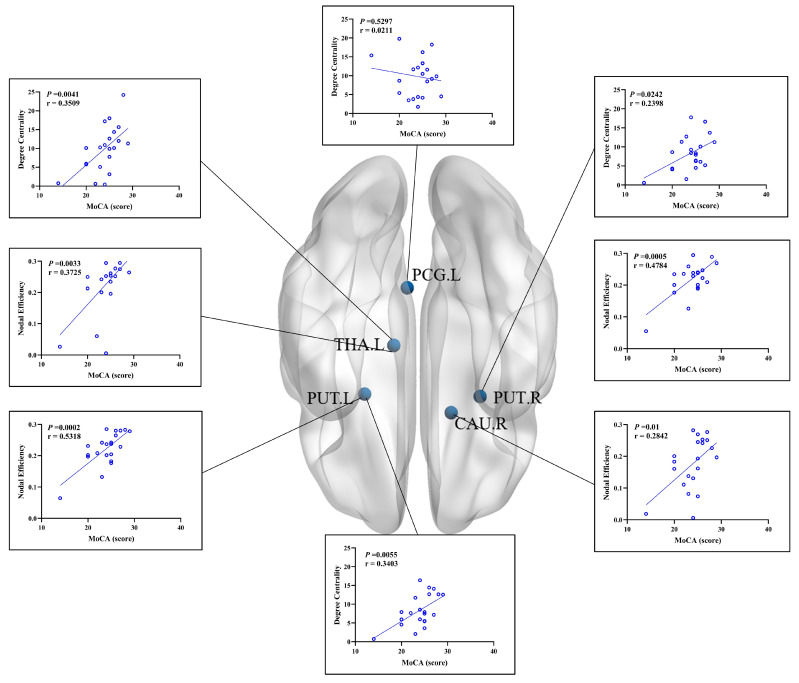
Scatter plot showing the correlation between node attributes and neuropsychological tests in CKD5 ND patients. PCG.L, posterior cingulate gyrus; THA.L, thalamus; CAU, caudate nucleus; PUT, lenticular nucleus, putamen; L, left; R, right; MoCA, Montreal Cognitive Assessment.

The correlation analysis between small-world attributes and MoCA scores in patients with CKD5 ND showed that MoCA scores were positively correlated with sigma and gamma values but negatively correlated with Lp scores. The correlations of MoCA scores with the topological attributes of the five nodes in the basal ganglia are shown in Figure 5. Node efficiency of the bilateral PUT (r = 0.53, *p* < 0.001 and r = 0.47, *p* < 0.001), left THA (r = 0.37, *p* < 0.001), and the right CAU (r = 0.28, *p* = 0.01) was positively correlated with the MoCA score. The MoCA score was positively correlated with the degree centrality of the left THA (r = 0.037, *p* < 0.05) and with the degree centrality of the bilateral PUT (r = 0.34, *p* < 0.05 and r = 0.23, *p* < 0.05 for the right and left PUT, respectively).

## 4. Discussion

This study applied a graph–theoretical approach to investigate the characteristic changes in the topology of functional brain networks of patients with CKD5 ND and HD as well as HCs. The results showed that the networks of all subjects exhibited small-world attributes. However, both patients with CKD5 ND and patients with HD displayed disrupted functional brain networks compared to HCs; this disruption was associated with CI. In particular, patients with CKD5 ND had more pronounced changes in small-world attributes than patients with HD, indicating a more severe disruption of functional brain networks in patients with CKD5 ND. The graph–theoretical analysis is helpful to reveal the pathological mechanism of CI in patients with CKD5 ND.

Small-world properties reflect an optimal equilibrium between the integration and separation characteristics of the network [30] that represent the efficient processing and transmission of information [31]. The brain networks of all subjects in this study had small-world properties, consistent with previous reports that the human brain is a small-world network with high clustering coefficients and a low Lp [32]. In the present study, CKD5 ND and HD patients demonstrated lower sigma and gamma values than HCs, suggesting that these patients have reduced small-world attributes, which supports the findings of a previous study [15]. The Lp is defined as the average of the shortest path between all pairs of nodes [5]. Small Lp values indicate that the network has a strong ability to integrate information [33]. The reciprocal of the Lp is the Eg; the Eg and Eloc reflect the ability of the network to transmit information at global and local levels, respectively [31]. In this study, patients with CKD5 ND and HD patients had significantly higher Lp values and significantly lower Eloc values than HCs. This finding indicates that the ability to integrate information and transfer information at the local level is reduced in these patients.

In this study, patients with CKD5 ND had significantly lower sigma and Eloc values than HD patients and significantly higher Lp values than HD patients. This suggests that CKD itself disrupts functional brain networks, with potential mechanisms including the accumulation of uremic toxins, reduced renal neurotrophic factors, and vascular damage to the central nervous system [2,34].

The putamen is an important nucleus in the basal ganglia, is involved in the formation of the striatum, and is associated with learning and motor control [35]. Previous studies have reported changes in the putamen in dialysis patients compared with HCs. Using quantitative susceptibility mapping (QSM) imaging, Chai et al. showed that the susceptibility to deposition in the bilateral putamen was higher in HD patients than in HCs and was significantly positively correlated with dialysis duration [36]. In addition, based on a graph–theoretical approach, Jin et al. found that the nodal efficiency of the putamen was greater in dialysis patients than in HCs [37], and Chou et al. showed that ESRD patients have a significantly lower nodal degree of the putamen than HCs [38]. All of these findings indicate that the connections with the putamen are altered in the brain network of ESRD patients. However, changes in the basal ganglia in patients with CKD5 ND are rarely reported. In our present study, CKD5 ND patients exhibited reduced centrality of the right putamen compared to the HC and HD groups, suggesting that CKD5 itself damaged local brain networks. Duygu et al. suggested that reduced putamen volume is associated with mild cognitive impairment [39], and Chai et al. reported that increased iron deposition in the gray matter of the putamen may be a risk factor for neurocognitive impairment [40]. Our study provides new evidence of cognitive impairment in patients with CKD5 ND, but further studies are needed to elucidate the underlying mechanisms.

In addition, we found that patients with CKD5 ND had lower nodal attributes in the left thalamus than in the HC and HD groups. Although some previous studies have reported changes in the thalamus of dialysis patients, Gu et al. found that volume reduction of the bilateral thalamus in HD patients [41] was correlated with MOCA scores, suggesting that thalamic volume reduction may be associated with cognitive impairment. Jin et al. reported that bilateral thalamic volume was increased in HD patients, accompanied by decreased thalamic-cortical network connectivity; however, reports of such change in patients with CKD5 ND are rare [37]. Wang et al. reported reduced connectivity between the thalamus and the rest of the brain in patients with mild cognitive impairment, possibly due to reduced integrity of the relevant cortical networks [42]. Therefore, we speculated that patients with CKD5 ND may have impaired brain network integrity, resulting in reduced functional connectivity of some brain regions. In this study, node attributes in some brain regions were increased, which we considered compensating for cognitive impairment.

In our study, the MOCA score of patients with CKD5 ND was lower than 26, indicating mild cognitive impairment [43], as reported in other comorbid cognitive disorders [44,45,46]. In addition, our results showed that the small-world properties and gamma values of patients with CKD5 ND were positively correlated with MOCA scores, while the Lp was negatively correlated with MOCA scores, suggesting that patients with CKD5 ND have diminished small-world properties of brain functional networks, reduced information transfer efficiency, and impaired functional networks, which may be related to cognitive impairment. We also found that the nodal efficiency of the bilateral putamen, left thalamus, and right caudate nucleus in patients with CKD5 ND were all positively correlated with MOCA scores, suggesting that abnormal basal ganglia circuitry in patients with CKD5 ND may be a potential mechanism contributing to cognitive impairment.

## 5. Limitations

We acknowledge that this study has some limitations. First, the relatively small sample size may have biased the results; thus, we will continue to increase the sample size in the future. Second, this study was a cross-sectional study. In the future, we will follow patients with CKD5 ND in an in-depth longitudinal study. Third, the AAL90 template was used in this study; however, results may differ depending on the template. In the future, we will use different templates to verify our findings. Finally, we used a single neuroimaging modality in our study. In the future, we will combine multiple modalities to further investigate the topographical characteristics of the brains of patients with CKD5 ND.

## 6. Conclusions

In summary, we used a graph–theoretical approach to analyze the brain topography of CKD5 ND and HD patients as well as HCs. Compared to the HC group, both CKD5 ND and HD patients showed reduced small-world attributes; patients with CKD5 ND also showed decreased Eloc. Patients with CKD5 ND had a more pronounced decrease in the small-world attribute. In addition, the areas with decreased nodal attributes in patients with CKD5 ND were mainly distributed in the basal ganglia. Importantly, decreased small-world attributes and decreased degree centrality of the basal ganglia may be the potential etiologies underlying cognitive impairment in patients with CKD5 ND. Overall, our findings reveal that patients with CKD5 may exhibit cognitive impairment before dialysis and that disruption of functional brain networks may be associated with this impairment; however, the underlying mechanisms need further investigation.

## Figures and Tables

**Table 1:** Demographic and clinical characteristics of the participants.

	CKD5 ND (*n* = 21)	HD (*n* = 32)	HCs (*n* = 32)	*p*-Value
Age (years)	48.9 ± 14.9 (21–76)	53.4 ± 9.7(32–66)	51.2 ± 10.3(30–65)	0.533 ^a^
Gender (male/female)	15/6	21/11	17/15	0.359 ^b^
Education (years)	11.9 ± 3.3 (5–20)	11.1 ± 3.5 (5–17)	12.3 ± 3.2 (6–19)	0.283 ^a^
HD duration (months)	NA	101.2 ± 21.3 (41–200)	NA	NA
Urea (mmol/L)	32.47 ± 9.15	20.34 ±4.57	NA	0.001 ^c^
Creatinine (μmol/L)	858.7.1 ± 317.6	391.3 ± 73.32	NA	0.001 ^c^
Phosphate (mmol/L)	2.06 ± 0.55	1.99 ± 0.52	NA	0.604 ^c^
Ca^2+^ (mmol/L)	2.09 ± 0.25	2.22 ± 0.25	NA	0.003 ^c^
Parathyroid hormone (pg/mL)	192.9 ± 146.2	237.8 ± 212.3	NA	0.375 ^c^
Hemoglobin (g/L)	94.3 ± 16.4	117.1 ± 12.2	NA	0.001 ^c^
Ferritin (ng/mL)	117.9 ± 121.3	199.6 ± 142.4	NA	0.055 ^c^
Serum iron (μmol/L)	14.1 ± 8.5	14.2 ± 5.2	NA	0.988 ^c^
MoCA scores	24.5 ± 4.1	22.4 ± 2.8	NA	0.041 ^c^

Data are presented as the mean ± standard deviation (range of min-max). CKD5 ND = nondialysis patients with stage 5 chronic kidney disease; HD = hemodialysis; HCs = healthy control; HD duration = hemodialysis duration; MoCA scores = Montreal Cognitive Assessment scores; NA = not applicable. ^a^ ANOVA. ^b^ Fisher’s exact test. ^c^ Two-sample *t*-tests.

**Table 2:** The abnormal node network characteristics displayed by pairwise comparison between the CKD5 ND, HD, and HC groups.

Brain Regions	Degree Centrality		Nodal Betweenness Centrality		Nodal Efficiency	
	t Values	*p* Values	t Values	*p* Values	t Values	*p* Values
CKD5 ND > HCs						
ORBsup.L	2.0886	**0.0418**	7.7503	0.4419	1.4607	0.1502
ORBsup.R	2.4089	**0.0197**	1.5116	0.1368	1.9220	0.0602
ORBmid.L	4.3239	**0.0001**	2.6948	**0.0095**	3.1636	**0.0026**
ORBmid.R	3.7782	**0.0004**	2.1214	**0.0388**	2.8810	**0.0058**
ORBinf.L	3.0087	**0.0041**	1.3395	0.1863	2.1590	**0.0356**
ORBinf.R	2.9957	**0.0042**	3.2584	**0.0020**	2.1744	**0.0343**
REC.R	1.9195	0.0605	2.1339	**0.0377**	1.1891	0.2399
CAL.R	1.8520	0.0698	2.6670	**0.0102**	1.5296	0.1323
CUN.R	2.1206	**0.0388**	2.9363	**0.0050**	1.7570	0.0849
SOG.R	1.2791	0.2067	2.0377	**0.0468**	6.4938	0.5190
PoCG.L	1.3888	0.1709	2.7223	**0.0088**	1.2125	0.2309
PCUN.L	2.1500	**0.0363**	2.6462	**0.0108**	1.3741	0.1754
PCUN.R	2.9052	**0.0054**	2.7126	**0.0091**	1.9510	0.0566
CKD5 ND < HCs						
SMA.R	−2.4378	**0.0183**	−2.8074	**0.0071**	−2.9118	**0.0053**
DCG.L	−2.0138	**0.0493**	−1.7628	0.0839	−2.4969	**0.0158**
HIP.L	−1.6497	0.1051	−8.8442	0.3806	−2.0812	**0.0425**
IOG.L	−2.1583	**0.0356**	−8.6682	0.3901	−2.3987	**0.0201**
FFG.L	−2.3614	**0.0221**	−2.2797	**0.0268**	−2.8041	**0.0071**
SMG.L	−1.4213	0.1613	−8.1156	0.4208	−2.0512	**0.0454**
CAU.L	−2.5745	**0.0130**	1.7117	0.8648	−3.2442	**0.0021**
CAU.R	−2.1955	**0.0327**	−1.4915	0.1420	−2.9940	**0.0042**
PUT.L	−1.9589	0.0556	−8.3522	0.4075	−2.4671	**0.0170**
PUT.R	−2.8171	**0.0069**	−1.3416	0.1857	−3.2004	**0.0024**
THA.L	−2.3232	**0.0242**	−1.3092	0.1963	−2.3190	**0.0244**
THA.R	−2.6136	**0.0117**	−1.2456	0.2186	−2.5073	**0.0154**
MTG.R	−7.6067	0.4504	−2.6950	**0.0095**	−1.4152	0.1631
ITG.L	−1.4478	0.1538	−2.7404	**0.0084**	−1.9122	0.0615
ITG.R	−1.3108	0.1958	−2.1112	**0.0397**	−1.7888	0.0796
CKD5 ND > HD						
OLF.L	2.0996	**0.0407**	1.7261	0.0904	1.7869	0.0799
CUN.R	1.5499	0.1274	2.0805	**0.0425**	1.3690	0.1770
SOG.R	1.3428	0.1853	2.5580	**0.0135**	1.1130	0.2709
ANG.L			2.0182	**0.0488**		
TPOsup.L	1.6052	0.1146	3.0474	**0.0037**	1.2585	0.2139
CKD5 ND < HD						
MFG.L	−2.2126	**0.0314**	−2.9424	**0.0049**	−2.6910	**0.0096**
MFG.R	−2.3390	**0.0233**	−3.4173	0.7340	−2.7524	**0.0082**
SMA.L	−1.6947	0.0962	−1.8227	0.0742	−2.1073	**0.0400**
HIP.R			−2.5164	**0.0150**	−3.6725	0.7150
MOG.L	−1.8222	0.0743	−2.0290	**0.0477**	−2.1516	**0.0362**
PUT.R	−2.1026	**0.0405**	−1.1212	0.2675	−2.4562	**0.0175**
THA.L	−2.2948	**0.0259**	−1.4876	0.1430	−2.3097	**0.0250**
MTG.R	−1.7756	0.0818	−2.0977	**0.0409**	−2.2811	**0.0267**

Brain regions showing significant between-group differences (*p* < 0.05, shown in bold font), in at least one of the three nodal network properties are exhibited in Table 2. Abbreviation: CKD5 ND, Nondialysis patients with stage 5 chronic kidney disease; HD, Hemodialysis patients; HCs, Healthy controls; R, Right; L, Left; ORBsup, Superior frontal gyrus; ORBmid, Middle frontal gyrus, orbital part; ORBinf, Inferior frontal gyrus, orbital part; REC, Gyrus rectus; CAL, Calcarine fissure and surrounding cortex; CUN, Cuneus; SOG, Superior occipital gyrus; PoCG, Postcentral gyrus; PCUN, Precuneus; SMA.R, Supplementary motor area; DCG, Median cingulate and paracingulate gyri; HIP, Hippocampus; IOG, Inferior occipital gyrus; FFG, Fusiform gyrus; SMG, Supramarginal gyrus; CAU, Caudate nucleus; THA, Thalamus; MTG, Middle temporal gyrus; ITG, Inferior temporal gyrus; OLF, Olfactory cortex; ANG, Angular gyrus; TPOsup, Temporal pole: superior temporal gyrus; MFG, Middle frontal gyrus; MOG, Middle occipital gyrus; MTG, Middle temporal gyrus; DCG, Median cingulate and paracingulate gyri; FFG, Fusiform gyrus; ITG, Inferior temporal gyrus.

## Data Availability

The data and materials of this study are available from the corresponding author by request.

## Abbreviations

Abbreviation	Full Name
CKD5 ND	non-dialysis patients with stage 5 chronic kidney disease
HD	hemodialysis
HCs	healthy controls
ESRD	end-stage renal disease
CI	cognitive impairment
ASL	arterial spin labeling
QSM	quantitative susceptibility mapping
rs-fMRI	resting-state functional magnetic resonance imaging
MoCA	montreal cognitive assessment
AAL	automated anatomical labeling
Lp	characteristic path length
Cp	clustering coefficients
gamma	normalized clustering coefficient
lambda	normalized characteristic path length
AUC	area under the receiver-operating characteristic curve
sigma	small-worldness
Eg	Global efficiency
Eloc	local efficiency
SMG	supramarginal gyrus
OLF	olfactory cortex
TPOsup	superior temporal gyrus pars temporal pole
DCG	Median cingulate and paracingulate gyri
SMA	supplementary motor area
IOG	inferior occipital gyrus
FFG	fusiform gyrus
CAU	caudate nucleus
PUT	lenticular nucleus, putamen
THA	thalamus
ORBsup	orbital superior frontal gyrus
ORBmid	orbital middle frontal gyrus
ORBinf	orbital inferior frontal gyrus
PCUN	precuneus
CUN	cuneus
MTG	middle temporal gyrus
ITG	inferior temporal gyrus
REC	right gyrus rectus
HIP	hippocampus
CAL	surrounding cortex
SOG	superior occipital gyrus
PoCG	postcentral gyrus
MOG	middle occipital gyrus
ANG	angular gyrus
PCG.L	posterior cingulate gyrus
